# Environmental Health Research Involving Human Subjects: Ethical Issues

**DOI:** 10.4137/EHI.S892

**Published:** 2008-07-14

**Authors:** David B. Resnik

**Affiliations:** Bioethicist National Institute of Environmental Health Sciences, National Institutes of Health, Research Triangle Park, NC

## Abstract

This article reviews some of the ethical issues that arise in environmental health research with human subjects, such as minimizing risks to subjects, balancing benefits and risks in research, intentional exposure studies with human subjects, protecting third parties in research, informing subjects about environmental hazards, communicating health information to subjects, and protecting privacy and confidentiality.

## Introduction

Since the U.S. adopted regulations governing research with human subjects in the 1970s, most of the ethical debates about research with human subjects have focused on questions relating to clinical research, such as management of risks, using placebos in control groups, randomization, informed consent, reporting adverse events, recruitment of subjects, and research on vulnerable populations. Most of the infamous or controversial cases discussed in the literature on human experimentation—the Nazi Experiments, the Tuskegee Syphilis Study, the Department of Energy’s secret human radiation experiments, and HIV research in developing nations—have been about medical research conducted in a clinical setting (Coleman et al. 2005; Emanuel et al. 2008; Levine 1988). In the last five years, however, ethical issues in environmental health research with human subjects have drawn greater attention (Resnik et al. 2005; Resnik and Zeldin, 2008; Resnik and Wing, 2007; Resnik, 2008; Resnik, 2006; Institute of Medicine, 2005; National Research Council, 2004; Sharp, 2003). Most of the ethical issues that occur in environmental health research are similar to those that arise in clinical research, but since there are important scientific, social, and political differences between environmental health research and clinical research, these familiar issues appear under a new light. This article will review some of the more challenging ethical issues in environmental health research.

## Environmental Health Research with Human Subjects

To understand some of the ethical issues in environmental health research with human subjects, it is important to distinguish between different research methods used by investigators, since these methods generate different ethical questions and problems. Environmental health research methods involving human subjects can be classified as either observational or experimental. Observational studies gather information about human subjects in their natural environment, whereas experiments gather information on human subjects under controlled conditions. Some of the designs commonly used in observational research include case-control studies, cohort studies, field studies, and cross-sectional studies. These study designs are commonly used in epidemiological and medical research. In a retrospective case-control study, investigators collect information on the environmental exposures of a group of people with a disease or condition (cases) over a period of time and a group of people similar to the cases but who do not have the disease or condition in question. In a prospective cohort study, investigators follow one group of people (the cohort) who have a particular characteristic (such as an environmental exposure) for a long period of time (10 years or more). Investigators also follow a comparison group of people (the control group) who do not have the characteristic. Field studies are similar to prospective cohort studies, except the observational period is much shorter: a field study may involve observation of subjects over a period involving days, weeks, or months. In a cross-sectional study, investigators make observations of a population at a single point in time. In all of these methods, investigators attempt to determine whether observed differences between the groups (e.g. cases vs. controls or cohort vs. control) are due to differences in environmental exposures (Gertsman, 2003).

Experimental research with human subjects includes intentional exposure studies and interventional studies in a particular environment, such as a home, workplace, hospital, or school. Intentional exposure studies expose human subjects to a safe dosage of an environmental agent, such as ozone, dust, or allergens, under controlled conditions, such as a laboratory.^2^ Intentional exposure studies can help investigators to obtain a better understanding of causal pathways from exposure to disease (National Research Council, 2004). Interventional studies expose a group of subject (the experimental group) to an environmental health intervention (such as lead abatement or educational materials). Interventional studies usually also include a control group that is not exposed to the intervention. The control group may be exposed to a different intervention or none at all (Institute of Medicine, 2005).

## Basic Requirements for Ethical Research

Over the last three decades, ethicists, researchers, and scholars have come to recognize some basic requirements for ethical research with human subjects (see Shamoo and Resnik, 2008; Emanuel et al. 2000 for further discussion):

1. Scientific validity: research should well-designed and executed; the use of human subjects must be necessary to answer scientific questions.
2. Social value: research should be expected to produce benefits for society.
3. Risk minimization: risks to human subjects should be minimized.
4. Benefit/risk justification: the expected benefits of the study to the subjects or society must outweigh the potential risks to the subjects.
5. Informed consent: research subjects (or their representatives) should give their informed consent to participate in research.
6. Protection of confidentiality and privacy: the confidentiality and privacy of subjects should be protected to the extent allowable by law.
7. Equitable subject selection: the selection of subjects must be equitable; there must be a sound scientific or moral justification for including subjects in research or excluding them from research.
8. Protection of vulnerable subjects: vulnerable subjects, such as children, prisoners, or mentally disabled adults, should be protected from harm or exploitation in research.
9. Data and safety monitoring: research should be monitored to protect subjects from harm and ensure the integrity of the data.
10. Independent review: an independent committee, such as an institutional review board (IRB), should review and oversee research.

These ethical requirements are embodied in various legal rules and policies, including the U.S. federal research regulations (e.g. 45 C.F.R. 46 and 21 C F.R. 50, 56), the Nuremberg Code, the World Medical Association’s Declaration of Helsinki, and the Good Clinical Practice Guidelines (Coleman et al. 2005). These ethical requirements apply to all types of research, including environmental health research. This review will discuss several issues relating to the application of these requirements to environmental health research.

## Benefits and Risks

One of the important ethical differences between observational and experimental studies in environmental health research is that observational studies usually impose fewer risks on research subjects than experimental ones, because observational studies collect data on people in their natural environment (Resnik and Wing, 2007). Observational studies usually impose risks on subjects that are not greater than the risks subjects would ordinarily encounter in daily life, which are defined as “minimal risks” under the federal research regulations (see 45 C.F.R. 46.102(i)). For example, a prospective cohort study of farm workers that asks subjects to complete a questionnaire and health survey every five years and provide 5 ml of blood for genetic analysis would impose few risks beyond the risk of loss of confidentiality, which could be minimized by taking protective measures, such as securing data and controlling access to it. A field study of pesticide applicators that measures pesticide residue on the subjects before and after the workday and also measures pesticide metabolites excreted in the urine would also impose few risks beyond the risk of the loss on confidentiality. Although the subjects would be exposed to pesticides at work, this exposure would have occurred even if they did not participate in the study, thus, it is not a risk imposed by the study. The study adds only the risks of collecting a urine sample and pesticide residue, completing surveys and questionnaires, and potential loss of confidentiality.

Experimental studies may impose risks on research subjects that are more than minimal. For example, the Human Studies Division of the Environmental Protection Agency (EPA) has an exposure laboratory for conducting studies on the effects of air pollution on the human respiratory system. Subjects in different experiments are exposed to ozone, automobile emissions, or other pollutants under controlled conditions. Scientists measure the subjects’ responses to these exposures, and monitor the subjects’ medical condition (EPA, 2008). Some experiments that expose subjects to pollutants involve a bronchoscopy to examine the airway and collect a small piece lung tissue for analysis (Arjomandi et al. 2005). A bronchoscopy is a medical procedure in which a physician inserts a tube (known as bronchoscope) into a patient’s airways through the nose or mouth. The patient is usually sedated. The risks of a bronchoscopy include bronchial spasms, difficulty breathing, bleeding, cardiac arrhythmias, infections, hoarseness, and a 0.1% to 0.01% chance of death, depending on the patient and the procedure (WebMD, 2008; Rose and Knox, 2007).

Imposing more than minimal risks on research subjects who are not expected to receive any medical benefits from the research can be ethically controversial. Since the subjects will receive little or no benefits from their participation, the research can be justified only if it is expected to yield important benefits for society, such as the development of new medical treatments.^1^ For example, Phase I trials of new drugs on healthy subjects usually involve risks that are more than minimal but offer the participants no benefits. The risks of any particular Phase I trial depend on the type of drug being tested, but there is often a small but very real chance of death, permanent injury, or disability. These studies can be ethically justified, according to most commentators, because they are a necessary step in the development of new drugs to treat diseases (Shamoo and Resnik, 2006).

More than minimal risk environmental health research experiments, such as the exposure studies mentioned above, do not usually benefit society by paving the way for new medical treatments. Nevertheless, one might argue that these studies can be justified if they are expected to yield important biomedical knowledge that will help to promote and protect public health and they are not expected to cause permanent harm to the participants (Resnik, 2006). For example, exposing human subjects to ozone may help researchers better understand how ozone impairs lung function, which could lead to changes in preventative health recommendations or air pollution regulations. To justify these experiments, it is essential for investigators to take precautions necessary to minimize risks to the subjects, such as using inclusion/exclusion criteria to disqualify potential subjects who have an increased risk for developing health problems when participating in lung function studies, careful monitoring of subjects during the testing period, follow-up with subjects after the testing period, using a data and safety monitoring committee to oversee the research, and implementing effective procedures for reporting adverse events (Resnik, 2006).

If experiments that expose research subjects to ozone can be ethically justified, what about experiments that expose subjects to pesticides? Some private companies have conducted pesticide experiments on human subjects, exposing people to minute amounts of chemicals that can be toxic or deadly in larger doses. Some commentators have argued that these experiments are unethical, because they do not offer society any important benefits. Companies designed these experiments in order to generate data to convince regulatory agencies to weaken pesticide registration rules (Environmental Working Group, 1998; Goldman and Links, 2004). Others argue that even if these particular experiments were not justified, some pesticide experiments could be justified if they are expected yield important public health benefits (such as stricter pesticide regulations) and they satisfy stringent scientific and ethical standards (Resnik and Portier, 2005; National Research Council, 2004). The ethical issues concerning pesticide testing on human subjects have not been resolved, and this remains a controversial topic in environmental health research.

In the fall of 2004 a field study designed and funded by the EPA and Centers for Disease Control and Surveillance (CDC), with financial support from the American Chemistry Council (ACC), named the Children’s Environmental Exposure Study (CHEERS), became ensnared in the controversy concerning pesticide experiments on human subjects (Resnik and Wing, 2007). The aim of the study was to observe children’s exposures to pesticides and other chemicals in the home. Families with high pesticide use were invited to participate in the study, which also would include a control group of families with low pesticide use. The investigators planned to monitor pesticide exposures during a series of 30 visits to the home over a two-year period. The protocol called for investigators to take surface wipe samples from around the home and urine samples from the children. They would also ask parents to record their children’s activities with a video camera and to keep records of pesticide use in the home. Parents were not required to start using pesticides to be in study, and they could remain in the study even if they decided to stop using pesticides. The investigators planned to warn parents about unsafe uses of pesticides and dangerous pesticide levels detected in the home or the children. Parents would receive a free video camera and $970 if they completed all of the study activities. The three IRBs that reviewed the study classified it as minimal risk (Resnik and Wing, 2007).

The study was initiated in the fall of 2004. Critics charged that CHEERS was an intentional exposure study that treated children like guinea pigs. They argued that the investigators were planning to conduct a controlled experiment in which parents would be asked to expose their young children to pesticides in return for a considerable sum of money. Critics also claimed that the study targeted low-income groups and that support from the ACC constituted an unacceptable conflict of interest (Organic Consumers Association, 2005). EPA officials and investigators were planning to revise the study to deal with these criticisms, but they were unable to convince politicians and the public that the study should go forward. Bowing to political pressure, the EPA cancelled the study in the spring of 2005 (Resnik and Wing, 2007). One of the important lessons from the demise of CHEERS is that environmental health researchers who are conducting observational studies should take steps to avoid creating the perception that their work intentionally exposes people to environmental agents, such as pesticides. The protocol and the informed consent document should be designed to clearly communicate to all parties that the research is observational, not experimental. Participants should not be asked to change their environmental exposures in order to be in the study or required to not change their exposures in order to remain in the study (Resnik and Wing, 2007).

Issues pertaining to benefits and risks have also arisen in research on environmental health interventions, such as studies of lead abatement or mold remediation (Resnik, 2008; Institute of Medicine, 2005). In a study that has received considerable attention due to a lawsuit related to the research, investigators from the Kennedy Krieger Institute at Johns Hopkins University conducted a controlled trial on the effects of different types of lead abatement on houses with lead paint located in Baltimore, NY. Twenty-five low-income families with young children were enrolled in the study. They were randomly assigned to one of three experimental groups or two control groups. The experimental groups included families living in homes that received different degrees of lead abatement, but less than the full amount of abatement, while the control groups included families living in lead-free homes or homes that had received the full amount of lead abatement. The goal of the study was to determine whether less than the full amount of lead abatement is an effective method of preventing health hazards related to lead exposure in the home. Many people living in homes with lead paint (or their landlords) could not afford the thousands of dollars required for full abatement (*Grimes v. Kenney Krieger Institute,* 2001).

One of the issues in the Kennedy Krieger study was whether it was ethical to not offer full lead abatement to all of the families living in homes with lead paint, since full lead abatement was the standard procedure for minimizing the health hazards of lead paint (Institute of Medicine, 2005). Some critics of the study argued that denying full lead abatement to subjects in the experimental groups was analogous to denying an effective treatment to patients in a clinical trial of a new medication (Spriggs, 2004). There is a consensus among medical ethicists that patients with a serious medical condition should not be denied and effective treatment when one is available, because clinical investigators have an ethical duty to provide their patients/subjects with the standard of care (London, 2000). Defenders of the lead abatement study responded to this charge by claiming that the study was different from a clinical trial, because the investigators were not physicians and the subjects were not their patients. Hence, the investigators did not have an obligation to provide all of the subjects with full lead abatement; they only had an obligation to avoid exploiting the subjects. The study was not exploitative because it benefitted the subjects, who received some lead abatement. The study also benefitted the community (Buchan and Miller, 2006). These are complex issues that bear further investigation and analysis. Although environmental health investigators are usually not physicians with a duty to promote their health of their patients/subjects, they should take steps to ensure that research subjects are not harmed and the benefits and burdens of research are distributed fairly (Resnik, 2008).

It is also important to note that some environmental health studies impose risks on people who are not directly involved in research, i.e. third parties. For example, an environmental health invention in the home may impose risks on people who are not research subjects, such as occupants of the home, or a study of agricultural workers may impose risks on farmers who employ the workers (Resnik and Sharp, 2006). Addressing risks to third parties in research is ethically controversial, because most regulations and guidelines do not address risks to third parties. The federal research regulations, for example, only mention obligations to minimize risks to research subjects (Resnik and Sharp, 2006). Since human research regulations do not mention risks to third parties, some writers have questioned whether researchers and IRBs should address these risks at all. Others have argued that there is an ethical obligation to address third party risks based on the notion that researchers should avoid causing harm. Hence, researchers and IRBs should address risks to third parties, when these risks can be identified and prevented (Resnik and Sharp, 2006).

## Sharing Information with Subjects

Informed consent requirements in environmental health research are very similar to those in clinical research. Investigators must inform subjects about the goals, methods and procedures used in research; benefits, risks, alternatives, confidentiality protections; the right to withdraw from research; and whom to contact for more information. Consent forms should be written in language that is understandable to the subject, and consent discussions should also take place under circumstances that minimize the potential for coercion or undue influence. Consent should be documented, except in circumstances that involve procedures in which consent is not normally documented or the main risk of the research is loss confidentiality and the consent document is the only information linking the subject to the research (Department of Health and Human Services, 2005).

One of the consent issues unique to environmental health research is informing subjects (or their families) about the risks present in their environment. When researchers collect data on hazards in the home or work environment, they have an obligation to inform subjects about those risks, because subjects need to know about these risks to make an informed choice (Institute of Medicine, 2005; Resnik and Zeldin, 2008). For example, investigators in the CHEERS study had planned to inform parents about dangerous pesticide exposures and unsafe pesticide practices. One of the allegations made by the plaintiffs in the Kennedy Krieger lawsuit was that the investigators did not inform the parents about dangerous lead levels in their children in a timely fashion (*Grimes v. Kennedy Krieger Institute,* 2001). Although clinical researchers inform subjects about study-related risks, they usually do not inform subjects about risks present in the subjects’ environment.

Investigators may be reluctant to inform subjects about environmental risks because the subjects may decide to not enroll in the study, drop out of the study, or take steps that would affect the data. For example, consider a study of pesticide use in the home. If a family participating in the study is informed about the risks of pesticide use, they may decide to stop using pesticides rather than enroll in a study of pesticide use, or they may decide to drop out of the study, or they may decide to decrease their use of pesticides. Although investigators should be prepared to deal with the decisions that subjects may make when they receive information, they should not withhold information from subjects or potential subjects to control their behavior. Investigators should design their research to compensate for subjects’ decisions in response to the information they receive. For example, investigators could ensure that enrollment is high enough to compensate for potential withdrawals, and that data is collected and analyzed in a way that changes in the subject’s behavior will not significantly affect the results. Investigators should also be prepared to counsel subjects about risks that are discovered in the environment and to make referrals, so that subjects can take steps to reduce these risks (Institute of Medicine, 2004).

During an inspection of a home, worksite, or other environmental setting, investigators may become aware of hazards that are not directly related to the study but are discovered while conducting the study, also known as incidental findings (Illes et al. 2008). For example, while inspecting a house for mold, an investigator may also detect unstable stairs in the basement, wasp nests in the attic, or other problems that could pose a risk to the occupants. Researchers must decide whether to disclose these incidental findings to the occupants of the home. An argument for disclosure is that researchers have an ethical obligation to protect research subjects (and third parties) from harm, including harm that is incidental to the research study. If an investigator smells a gas leak in the basement during a mold inspection, it would be callous and irresponsible not to inform the occupants of the home about the leak. An argument against disclosure is that investigators may not have the knowledge or expertise to assess or discuss findings that are unrelated to their research. Additionally, subjects also do not expect to receive reports of such findings. Most environmental health researchers are not trained as electricians, plumbers, or home inspectors. One suggested solution to the dilemma concerning incidental findings is to disclose only those findings that a reasonable person would disclose under the circumstances, rather than the findings that an expert would disclose (Resnik and Zeldin, 2008).

Other types of information that investigators may consider sharing with research subjects include the results of tests conducted as part of the study, such as blood tests or genetic tests. The main reason to share these results with subjects is that they may contain information they can use to protect their health. For example, a parent would want to know if her child has elevated lead levels in her bloodstream, because she could use that information to decide whether her child should see a physician or whether she should take steps to reduce the child’s exposure to lead. Likewise, a family would want to know if their house contains an abnormally high amount of mold, because they could use that information to reduce mold levels.

The argument against sharing results from test conducted for research purposes is that they may be useless to the subjects or even uninformative. For example, researchers who are conducting a study to determine whether there are genetic variants that increase susceptibility to the adverse effects of mercury exposure may not know whether the information about genetic variants will be useful to subjects in making medical decisions. The data may be useful in discovering associations between genes and diseases or generating hypotheses, but not useful in making medical decisions. Subjects who learn about these test results find the information to be useless or even confusing. According to some authors, the results of tests conducted for research purposes should be returned to subjects only if the tests are reliable and accurate, and the information is likely to useful to subjects in making medical decisions (Renegar et al. 2006).

## Privacy and Confidentiality

Most of the confidentiality and privacy issues in environmental health research with human subjects are similar to those that arise in clinical research. For example, environmental health researchers should restrict access to data, store data in a secure place using secure methods, and remove information from the data that identifies individuals prior to publication. Investigators may remove all personal identifiers from the data if this information is not needed for research purposes. Investigators should also inform subjects about the measures that will be taken to protect their privacy and confidentiality (Shamoo and Resnik, 2008).

Some confidentiality and privacy issues are unique to environmental health research. When environmental health researchers conduct field studies in homes, businesses, or other venues, they may gain access to private information that is not part of the study (Institute of Medicine, 2005). For example, investigators conducting a study of allergen reduction in the home may find out about drug or alcohol use, sexual activity, health or psychological problems, and so on. Investigators should protect the privacy of their subjects and keep this information in confidence, unless they discover evidence of abuse of a child or vulnerable adult. Investigators should also inform subjects about their obligation to report evidence of the abuse of a child or vulnerable adult during the consent process (Resnik and Zeldin, 2008). In general, investigators should use discretion and good judgment when collecting data in the home, to avoid unnecessary disclosures of private information.

## Conclusion

This review has highlighted some of the ethical issues that arise in environmental health research with human subjects. There are of course some important issues that this review has not covered in depth, including conflicts of interest, payments to subjects, consulting with communities, and research with vulnerable populations.^3^ As one can see from this brief survey, the ethical issues that arise in environmental health research with human subjects are similar to those that arise in clinical research, but there are some situations unique to environmental health research that create dilemmas rarely encountered by clinical researchers. Many of these novel ethical dilemmas occur when investigators collect data or conduct interventions in the home environment or intentionally expose human subjects to dangerous environmental agents, such as pesticides. It is likely that other novel issues will emerge in environmental health research. Environmental scientists—and ethicists—should prepare to deal with them as they arise.

## Footnotes

1. Although subjects usually receive some payment for risk research that does not provide any medical benefits, most government agencies do not consider money to be a benefit. See Shamoo and Resnik (2006).

2. A “safe” dosage would be one that is not expected to cause any permanent harm to the research subject. Some substances that are harmful in larger doses, such as ozone, may cause little harm in very small doses. Some substances may cause permanent harm even in small doses. For example, known carcinogens, such as benzene and asbestos can cause permanent damage at very small exposures. It makes no difference whether the unsafe expose might occur in normal life. For example, a construction worker might inhale asbestos dust when helping to demolish an old building, but this would not justify intentionally exposing a human subject to inhaled asbestos. For further discussion, see Resnik (2006), National Research Council (2004).

3. For further discussion of conflicts of interest in biomedical research and research with vulnerable populations, see Shamoo and Resnik (2008); for discussion of payments to subjects, see Grady (2005); for discussion of community consultation in research, see Weijer and Emanuel (2000).
